# Monitoring Neurochemistry in Traumatic Brain Injury Patients Using Microdialysis Integrated with Biosensors: A Review

**DOI:** 10.3390/metabo12050393

**Published:** 2022-04-26

**Authors:** Chisomo Zimphango, Farah C. Alimagham, Keri L. H. Carpenter, Peter J. Hutchinson, Tanya Hutter

**Affiliations:** 1 Division of Neurosurgery, Department of Clinical Neurosciences, University of Cambridge, Cambridge CB2 0QQ, UK; fca21@cam.ac.uk (F.C.A.); klc1000@cam.ac.uk (K.L.H.C.); pjah2@medschl.cam.ac.uk (P.J.H.); tanya.hutter@utexas.edu (T.H.); 2 Walker Department of Mechanical Engineering, The University of Texas at Austin, Austin, TX 78712, USA

**Keywords:** biosensors, traumatic brain injury, neurochemistry, cerebral microdialysis, cerebral metabolism

## Abstract

In a traumatically injured brain, the cerebral microdialysis technique allows continuous sampling of fluid from the brain’s extracellular space. The retrieved brain fluid contains useful metabolites that indicate the brain’s energy state. Assessment of these metabolites along with other parameters, such as intracranial pressure, brain tissue oxygenation, and cerebral perfusion pressure, may help inform clinical decision making, guide medical treatments, and aid in the prognostication of patient outcomes. Currently, brain metabolites are assayed on bedside analysers and results can only be achieved hourly. This is a major drawback because critical information within each hour is lost. To address this, recent advances have focussed on developing biosensing techniques for integration with microdialysis to achieve continuous online monitoring. In this review, we discuss progress in this field, focusing on various types of sensing devices and their ability to quantify specific cerebral metabolites at clinically relevant concentrations. Important points that require further investigation are highlighted, and comments on future perspectives are provided.

## 1. Introduction

Traumatic brain injury (TBI) is a health burden affecting over 50 million people each year globally [1,2]. Its long-term adverse effects to the brain may create a significant burden implicating social integration, independence, and employability. Population-based studies have reported that 238,000 people each year sustain a TBI in the UK, and it is the overall leading cause of death in young adults (25–35 years) in developed countries [3]. In 2013, TBIs in the USA led to about 2.5 million emergency department visits, 282,000 hospitalisations, and 56,000 deaths [4]. Mild or severe TBI patients are at risk of secondary brain injury that arises from complex physiological responses to the initial injury. These complex physiological processes are thought to include a series of sequential events including altered cerebral metabolism. Monitoring cerebral metabolism along with other protocols to identify early changes and prevent secondary brain injuries during neurocritical care management can improve outcomes of severe TBI patients [5,6]. Currently, cerebral metabolism is monitored using cerebral microdialysis, an invasive technique in which interstitial brain fluid can be sampled. The sampled fluid (also termed microdialysate) is usually analysed hourly offline using a bedside analyser, for which its operating principle is based on enzymatic reactions [7,8]. However, the data analysed are retrospective, hindering real-time interventions. Thus, effective and timely measurements of the microdialysates are required to predict the onset of secondary brain damage. To fulfil this, emerging research has integrated microdialysis with biosensors for continuous online detection of relevant cerebral metabolites found in microdialysates. The intent of these technologies is to provide accurate and actionable information that may help inform clinical decision making and aid in the prognostication of patients’ outcome.

In this review, the state of emerging biosensors coupled with microdialysis technology in monitoring cerebral metabolism of TBI is presented and discussed. We start by explaining the broad overview of TBI and how it alters cerebral metabolism, followed by a description of the cerebral microdialysis principle and its clinical usefulness. Recent developments in novel biosensors to monitor brain metabolism are presented, and their performances in quantifying relevant brain metabolites are also discussed. We conclude by discussing the need for the further development of novel biosensors for direct in vivo real-time monitoring of brain metabolism.

## 2. Importance of Monitoring Brain Metabolism for TBI

### 2.1. Traumatic Brain Injury (TBI)

TBI is an insult to the brain due to an external force causing damage to the brain’s structure and function [9]. It represents mild, moderate, and severe effects of physical assault to the brain that may cause sequential, primary, or secondary ramifications. Thus, TBI can be classed into a primary mechanical injury, which is non-amenable to medical treatment and a delayed secondary brain injury involving various changes at the cellular and molecular level that could in theory be counteracted. The sudden mechanical injury is a widespread tearing, shearing, or stretching of axons, referred to as axonal injury, as well as contusions, hemorrhages, and lacerations [10]. Delayed secondary injury evolves hours or days after the initial primary mechanical trauma due to neuronal and glial dysfunction, neuroinflammation, cerebral oedema, and metabolic changes. Consequently, this leads to various physiologic alterations including hypoperfusion, blood–brain barrier (BBB) disruption, oxidative injury, and mitochondrial dysfunction [11,12,13,14,15,16]. This complexity of secondary brain injury poses diagnostic and therapeutic challenges; therefore, additional invasive monitoring interventions are required during neurocritical care of severe TBI patients. These additional metrics may provide insights into brain tissue oxygenation, intracranial pressure (ICP), cerebral perfusion pressure (CPP), electrophysiology, and local brain metabolism [11]. Abnormal local brain metabolism is linked to poor patient clinical outcomes, thus interrogating the brain to monitor its chemistry is necessary [17].

### 2.2. Cerebral Metabolism

**Figure 1:**
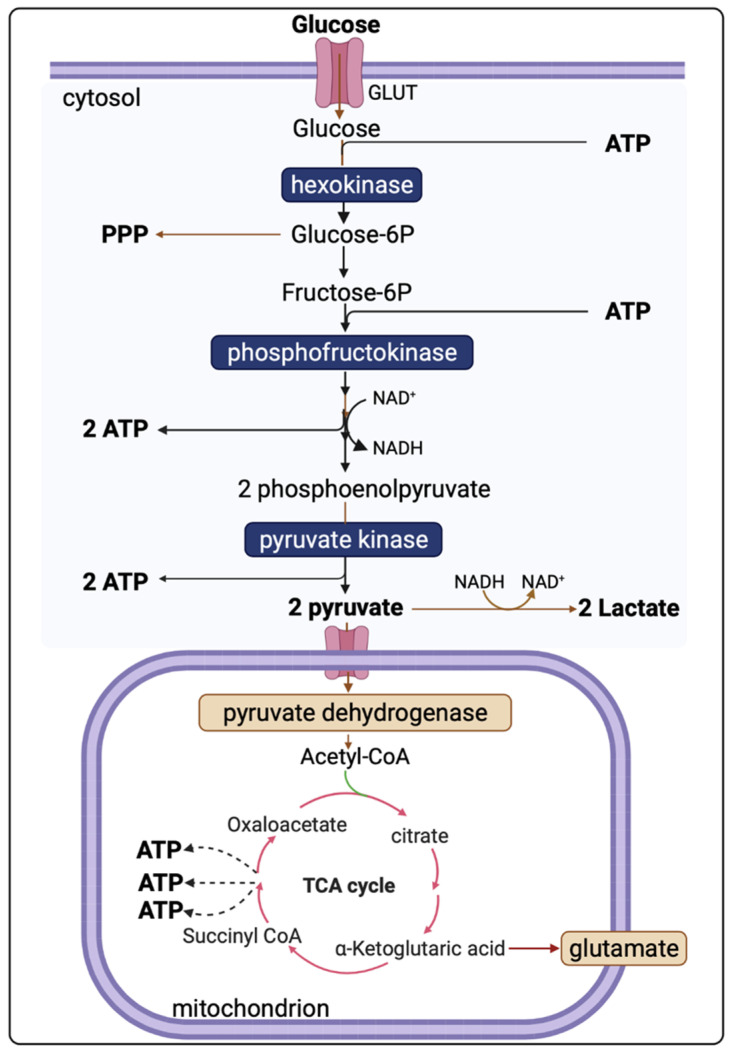
Illustration of glucose, lactate, and pyruvate within the major energy pathways. Hexokinases uses ATP to phosphorylate glucose to glucose 6P (glucose 6-phosphate) in the first irreversible step of glycolysis. Glucose 6P can either undergo alternative metabolic fates or continue down the glycolytic pathway to generate pyruvate. Pyruvate can be utilised either by oxidative metabolism via the tricarboxylic acid (TCA) cycle or remain in the cytosol where it is converted to lactate. The glycolytic pathway that takes place in the cytoplasm produces a net yield of 2 ATP per molecule of glucose (2 ATP molecules are utilised early on but then paid back later with the generation of 4 ATP). Overall, the yield per mole of glucose metabolised fully to CO_2_ by combination of glycolysis and mitochondrial metabolism is theoretically 36–38 moles of ATP.

Evidence shows that cerebral metabolism is disturbed following TBI, although the exact mechanisms are incompletely understood due to the complex and heterogeneous nature of TBI [7]. The brain uses glucose as a preferred substrate for energy consumption, so the regulation of cerebral glucose metabolism is crucial. Oxidative metabolism of glucose provides most of the ATPs utilised by the brain. However, biosynthetic routes that branch from the glycolytic pathway and the tricarboxylic acid (TCA) cycle and other pathways including the pentose phosphate shunt, glucose storage as glycogen, and the malate-aspartate shuttle all have significant roles [18]. Understanding of the altered cerebral metabolism is incomplete without knowledge of the glucose metabolism in an uninjured brain. Figure 1 presents the major energy pathways in the brain.

In the uninjured brain, glucose uptake by both neuronal and glial cells undergoes oxidation via glycolysis to form two pyruvate molecules. Glycolysis is independent of oxygen and, thus, can occur under either aerobic or anaerobic conditions. The energy yield of ATP per molecule of glucose depends on whether mitochondrial shuttle mechanisms are operational or not. To generate one molecule of pyruvate by glycolysis, one molecule of NAD^+^ is converted into NADH, which must be recycled (oxidised) back to NAD^+^ if glycolysis is to be sustained. One recycling mechanism is by the electron-transport chains of mitochondria (if operational). As NADH cannot cross the mitochondrial membrane, the requisite hydrogens and electrons are transferred indirectly by “shuttle” mechanisms-malate-aspartate shuttle system and/or the glycerol-3-phosphate shuttle [19]. NADH can also be recycled to NAD^+^ by the lactate dehydrogenase (LDH)-mediated conversion of pyruvate to lactate in the cytosol, as an epilogue to “anaerobic” glycolysis. 

The overall reaction of glycolysis and net yield of ATP molecules is presented in Equation (1).

(1) $$Glucose+2ADP+2P_{i\;}+2NAD^{+}\to 2pyruvate+2ATP+2NADH+2H^{+}+2H_{2}O\;$$

Pyruvate, the end-product of glycolysis, can enter mitochondria where it is converted to acetyl-CoA by pyruvate dehydrogenase. Acetyl-CoA is further metabolised in the tricarboxylic acid (TCA) cycle in mitochondria. The sum of all the reactions in the TCA cycle is presented in Equation (2).

(2) $$Acetyl-CoA+3NAD^{+}+3FAD+GDP+P_{i}+2H_{2}O\to CoA-SH+3NADH+FADH_{2}+3H^{+}+GTP+2CO_{2}\;$$

Subsequently, NADH and FADH_2_ are utilised by the mitochondrial electron transport chain (by Complexes I and II, respectively); electrons are transferred to complexes III and IV, where O_2_ is the terminal electron acceptor on Complex IV, followed by ATP synthesis by ATP synthase (also termed Complex V) [20]. The yield per molecule of glucose metabolised fully to CO_2_ (by combined glycolysis, NADH shuttling, and mitochondrial respiration) is theoretically 36–38 ATP molecules. However, the actual yield is considered somewhat lower [21,22].

### 2.3. Altered Cerebral Metabolism Due to TBI

Imbalance in cerebral glucose metabolism following TBI is well-documented [7,23,24] and attributable, at least partly, to the altered ATP production in the brain’s major energy pathways. Due to a high energy demand following TBI, abnormally low levels (<0.8 mM) of extracellular glucose occur [17], possibly because of upregulated glucose uptake by neurones and glia. Conversely, neurones and glia may sometimes be too damaged to take up glucose from extracellular fluid, leading to hypometabolism characterised by abnormally high extracellular glucose. Thus, there is an optimum extracellular glucose range, although there is insufficient evidence to define this exactly [25]. Extracellular lactate can also be utilised as an alternative fuel [24]. ^13^C-labelled microdialysis studies have demonstrated that the traumatically injured brain uses lactate via the TCA cycle [24,26]. Another ^13^C-labelled microdialysis study found lactate production from 1,2-^13^C_2_ glucose via glycolysis and to a lesser extent via PPP [23]. Lactate was also identified as a spin-out product (cataplerosis) from the TCA cycle in ^13^C-labelled microdialysis studies using 2,3-^13^C_2_ succinate as a substrate [11,23].

A persistent high lactate/pyruvate ratio (LPR) (LPR > 25 or >40) indicates metabolic dysfunction or crisis. In a microdialysis study of 233 TBI patients, acute-phase LPR > 25 was associated with poor clinical outcomes 6 months later [17]. High LPR, despite seemingly adequate oxygen and glucose delivery to brain tissues, is regarded as indicating mitochondrial dysfunction [11,27]. The concentrations of lactate and pyruvate and their ratio (LPR) provide useful information about the cellular redox state in the region of interest. The extracellular LPR is thought to reflect the LPR in the cytoplasm—itself in equilibrium with cytoplasmic NADH/NAD^+^ ratio [28]. Glucose, lactate, pyruvate, and LPR were cited as the most clinically relevant biomarkers in a consensus statement from the 2014 International Microdialysis Forum [25]. Timely assessment of these is, therefore, essential in the early detection of secondary brain injury allowing prompt interventions. Table 1 summarises neuroprotective interventions for altered neurochemistry.

## 3. Review of Sensor Technologies for Brain Metabolism

In selecting scholarly articles to discuss in this review, emphasis was placed on work published in the areas of biosensors, TBI, cerebral metabolism, and microdialysis in the last 10 years. We first discuss the current routinely used method and then present others. Table 2 summarises some of the most relevant articles discussed in this review. Those biosensors are in the research and development phases and are not approved for routine clinical use.

### 3.1. Cerebral Microdialysis

**Figure 2:**
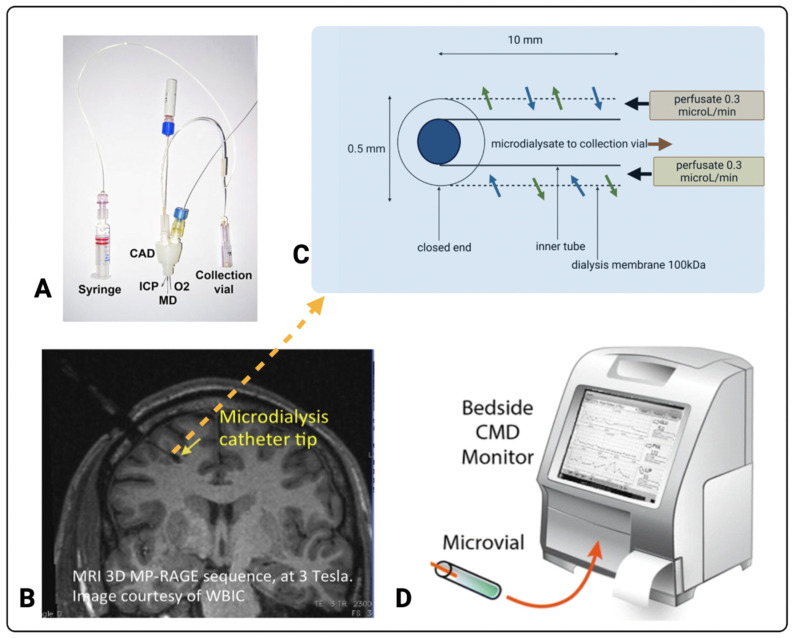
Cerebral microdialysis. (**A**) Triple lumen cranial access device (CAD) that allows microdialysis (MD), intracranial pressure (ICP), and brain tissue oxygen (O_2_) probes through the skull into the brain (image copyright K.L.H. Carpenter, reproduced with her permission). A syringe for supplying perfusion fluid and a microdialysate collection vial are also shown. A pump (not shown) drives the syringe that delivers perfusion fluid into the microdialysis catheter. The microdialysis catheter tip is typically placed in the white matter of the right frontal lobe, as shown in the magnetic resonance image (MRI) (**B**) (reproduced from [7]). (**C**) The architecture of the microdialysis catheter tip (adapted from [53]). The microdialysates are assayed offline, typically hourly, using the ISCUSflex bedside analyser (**D**) (image reproduced from [11]) for glucose, lactate, and pyruvate and often also for glutamate and glycerol. Images (**A**,**B**,**D**) originally appeared in [53], [7], and [11] respectively, published Open Access CC BY, copyright The Authors.

The use of cerebral microdialysis during neurocritical care management of TBI patients along with other metrics such as ICP and PbtO_2_ aims at minimising or preventing the burden of secondary brain damage [5,25,27]. Cerebral microdialysis is a neuromonitoring tool that reflects brain-tissue health and metabolism [52]. A microdialysis probe perfused with physiological salts solution is placed in the brain’s parenchymal region, allowing sampling of molecules from the brain’s extracellular fluid. The cerebral microdialysis technique including its components is represented in Figure 2.

In a clinical setting, cerebral microdialysis is used along with ICP and PBtO_2_ sensors and is a part of multi-modality monitoring enabled by a triple lumen cranial access device [54] (Figure 2A), which holds the three probes in the brain parenchyma (Figure 2B). Typically, CNS perfusion fluid (M Dialysis AB, Stockholm, Sweden), containing 147 mM NaCl, 2.7 mM KCl, 1.2 mM CaCl_2_, and 0.85 mM MgCl_2_ in ultrapure water, is pumped (using a syringe pump) at a low flowrate (typically 0.3 µL/min) through the inlet tubing and down the outer tube of the catheter to the distal end where the last 10 mm (or in some cases 20 mm or 30 mm) of the outer tube is composed of a semi-permeable membrane, where the bi-directional exchange of molecules occurs by diffusion between the fluid in the catheter and the brain’s extracellular interstitial space [8]. The catheter tip has a closed end, allowing the return of the perfusion fluid—now termed microdialysate—up the inner tube of the catheter, and it is collected in a micro-vial. A schematic of the microdialysis catheter tip is shown in Figure 2C. The semi-permeable membrane typically used clinically has either a 20 kDa or 100 kDa nominal molecular weight cut-off. The microdialysate contains the molecules of interest, including markers of brain metabolism such as glucose, lactate, and pyruvate. Mean relative recoveries (%) (also known as extraction efficiency) using 20 kDa or 100 kDa catheters at a flow rate of 0.3 µL/min have been determined in vitro as 94–105% for glucose, 87–95% for lactate, and 92–105% pyruvate [52].

For clinical monitoring, the collected microdialysate in the microvial is typically analysed hourly, using the standard enzymatic colorimetric bedside analyser (ISCUSflex clinical microdialysis analyser, M Dialysis AB, Stockholm, Sweden), which is shown in Figure 2D. Timely assessment of these metabolites is essential in the early detection of secondary brain injury, allowing prompt interventions. Therefore, the development of novel biosensors for neurochemical monitoring is centred around the rapid and continuous detection of the relevant metabolites in TBI patients. 

### 3.2. Current Standard for TBI Monitoring in the ICU

The concentrations of glucose, lactate, and pyruvate in patients’ brain extracellular fluid samples retrieved through microdialysis have been determined through a widely used ISCUSflex Microdialysis Analyser (M Dialysis AB, Stockholm, Sweden). The commercially available ISCUSflex analyser and, from the same manufacturer, earlier models, ISCUS and CMA600, are examples of enzymatic colorimetric analysers. Glucose, lactate, and pyruvate are each enzymatically oxidised by reagents that contain glucose oxidase, lactate oxidase, and pyruvate oxidase, respectively, producing hydrogen peroxide as a by-product. The reagents also contain peroxidase and dye-precursors, and when they react with the hydrogen peroxide, this leads to the formation of red-violet-coloured products: quinoneimines for glucose and lactate, and a quinonediimine for pyruvate. The rate of formation of these coloured products is proportional to concentrations of each analyte. Glucose, lactate, and pyruvate are assayed sequentially on discrete small aliquots of microdialysates (0.5, 0.2, and 0.5 mL, respectively). ISCUSflex, ISCUS, and CMA600 are advantageous due to their ability to handle small microdialysate volumes and have low limits of detection of 0.1 mM for both glucose and lactate and 10 mM for pyruvate. The ability of the ISCUSflex, ISCUS, and CMA600 to produce absolute values of the three clinically relevant metabolites along with LPR has proven useful in microdialysis-based studies where findings have been correlated with clinical outcomes [17,27]. Though the ISCUSflex, ISCUS and CMA600 satisfy the clinical requirements with respect to sensitivity and selectivity, and they are hampered by the need for fresh reagents every few days and these are expensive. They also require hourly analyses by clinical staff, and, therefore, are very labour-intensive. On a busy shift, clinical staff are sometimes unable to analyse microdialysates in a timely manner. The limitation of hourly—or even less frequent—readings lead, in effect, to retrospective metabolic assessments that are less useful to making clinical judgements. These drawbacks highlight the need for devising biosensing techniques for online continuous monitoring of brain metabolism in TBI patients during neurocritical care. It should be noted that ISCUS and CMA600 are no longer marketed; ISCUSflex (Figure 2D) is the only clinical enzymatic colorimetric microdialysis analyser currently available commercially. 

### 3.3. Introduction to Biosensors

By design, any biosensor comprises (1) an element that detects the substrate of interest and produces a response signal, (2) a transducer, which transforms the generated response into a detectable response, and (3) a detector capable of enhancing and processing signals before they are displayed on an electronic digital platform [55]. It is noteworthy that the field of biosensors is very broad, and their categorisation in the literature is not straightforward. However, based on a transducer, biosensors can be classified as electrochemical, optical, and others [55,56,57,58].

**Figure 3:**
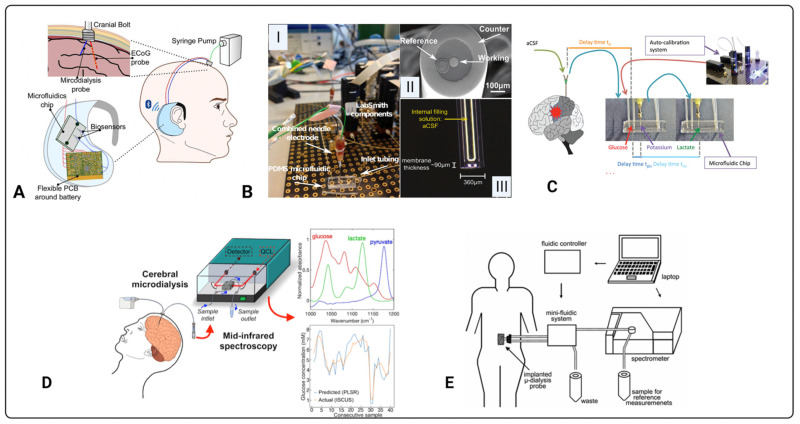
Biosensors coupled to microdialysis for quantification of clinical analytes. (**A**) Proof-of-concept design for a wireless, portable bio-instrument to monitor neurochemicals in brain injured patients [47]. Previous work from the same group developed a biosensor setup (**B**): (**I**) is the combined needle with a PDMS microfluidic chip placed within a LabSmith automated platform for glucose detection, (**II**) tip of needle electrode, and (**III**) potassium ion selective electrode [45]. Similarly, (**C**) shows a setup as in (**B**) but includes a lactate biosensor and demonstrates integration with microdialysis in-vivo [50]. (**D**) Illustration of an optical based sensor integrated with brain microdialysis for brain metabolite monitoring [49]. (**E**) is another optical based biosensor set up for glucose monitoring in the dialysates of subcutaneous interstitial fluid [66]. Images (**A**–**D**) originally appeared in [45,47,49,50], respectively, published Open Access CC BY, copyright The Authors. The diagram in (**E**) is published with permission of the Publisher and Corresponding Author*; it originally appeared in H. M. Heise*, V. R. Kondepati, U. Damm, M. Licht, F. Feichtner, J.K. Mader, and M. Ellmerer. Microdialysis based monitoring of subcutaneous interstitial and venous blood glucose in type 1 diabetic subjects by mid-infrared spectrometry for intensive insulin therapy. Optical Diagnostics and Sensing VIII, edited by G.L. Cote, A.V. Priezzhev. Proc. of SPIE Vol. 6863, 686308, (2008), 1605–7422/08/$18, doi:10.1117/12.772050 [66].

Ever since Clark and Lyons introduced a biosensor to monitor blood gas levels in 1962 [59], recent advances have facilitated alternative detection modalities. Since then, various types of biosensors for specific detection of analytes [60,61,62] have been developed; hence, this is now a multidisciplinary area bridging fundamental science (chemistry, physics, and biology) and clinical practice. An early example of a biosensor integrated with microdialysis for diagnostics was reported by Roda and colleagues in 1991 to monitor extracellular lactate in a rat brain [63]. The enzyme-based electrochemical biosensor showed a high performance in quantifying lactate attributed to a limit of detection (LoD) of 0.1 mM, a wide linearity range between 0.1 and 100 mM, and an imprecision of less than 5%. Advantageously, this biosensor quantified other small molecules including glycerol. Since then, numerous biosensors coupled to microdialysis for the quantification of molecules found in body fluids, tissues, and organs other than the brain have been reported [64,65,66,67,68,69,70,71,72,73,74,75,76,77,78]. An earlier review of biochemical monitoring of various bodily fluids has been presented elsewhere [79]. Examples of biosensors integrated with microdialysis for analyte detection are presented in Figure 3.

Neurochemical changes in TBI may also be propagated by an occurrence of spreading depolarisations, which originate from the lesion foci and spread out to neighbouring tissues at risk of secondary brain damage. This causes a dramatic disruption of ionic homeostasis, leading to a dramatic increase in extracellular potassium ions [80,81], neuronal and astrocytic swelling [82,83], glucose depletion and accumulation of lactate leading to tissue acidosis [84,85,86,87], and changes in cerebral blood flow [88]. The characteristic features of spreading depolarisations are a large transient negative shift in the slow electrical or direct current potential and the simultaneous silencing of brain electrical activity termed spreading depression [89,90]. This has led to the development of electrochemical-based potassium sensors integrated with microdialysis. An in-depth discussion of potassium biosensors and potassium measurements is beyond the scope of this review. However, their relevance to the field, especially when potassium measurements are interpreted along with neurochemical measurements, cannot be overlooked and have been briefly mentioned in Section 3.4.

A very large body of work on continuous monitoring of microdialysates using electrochemical detection has been carried out by Boutelle and colleagues. Notable examples of their recent original research relevant to acute brain injury include work by Papadimitriou et al. (2016), Rogers et al. (2017), Robbins et al. (2019), Gowers et al. (2019), Tageldeen et al. (2020), and Gifford et al. (2021), which are summarised in Table 2. The use of various types of biosensors in clinical research is growing. Figure 3 highlights the diversity of the biosensor technologies and their respective setups in tandem with microdialysis. For instance, Figure 3A–D all have measured cerebral metabolites in dialysates of TBI patients, while Figure 3E has quantified blood in the dialysates of subcutaneous interstitial fluid [45,47,49,50,65]. The development of biosensors in monitoring cerebral metabolism is challenging, and this is evident as no biosensor has been approved for clinical use. The complexity and heterogeneity of TBI as well as the difficulty in fabricating ultrasensitive sensors for monitoring brain metabolism are factors slowing the progress. The development of a clinically effective biosensor requires optimisation and validation [55]. For effective optimisation and validation, the sensor must satisfy several factors including selectivity, sensitivity, stability, reproducibility, linearity, and multiplexing [55]. For ease and clear communication, each parameter has been defined in Table 3 in the context of brain metabolism in TBI. The most common types of sensors developed for metabolite monitoring in TBI are electrochemical or optical, and they are thoroughly discussed in the next sections.

### 3.4. Electrochemical Sensors

Electrochemical sensors are widely used for the detection of metabolites owing to their fast, precise, selective, and easy-to-use capabilities. They operate by producing an electrical signal proportional to the concentration of the analyte of interest [55]. Generally, chemical sensors rely on conductometric, potentiometric, or amperometric measurements [91]. 

**Figure 4:**
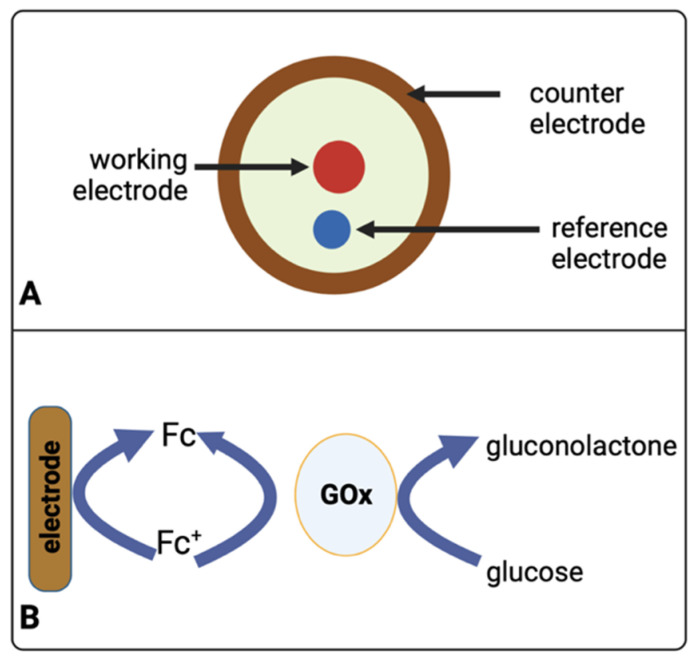
(**A**) Schematic diagram of a three-electrode system (biosensor) used to sense glucose. The schematic presented here for clarity is also a representation of the image Figure 3B(II). (**B**) Oxidation of glucose at an electrode mediated by a ferrocene derivative (Fc). Glucose oxidase (GOx) is an enzyme that catalyses glucose oxidation.

In principle, most commonly used biosensors utilise a three-electrode system (Figure 4A) consisting of a working electrode (WE), a reference electrode (RE), and a counter electrode (CE). Figure 4B schematically shows how enzymes play an important role in electrochemical detection, whereby an electrical current arising during oxidation (reduction) of glucose on the surface of a working electrode, under certain potentials, is measured. Electrochemical methods for analysing glucose typically use the enzyme glucose oxidase (abbreviated as GOx or GOD in the literature) to catalyse the conversion of glucose to gluconolactone. In some assay formats, the oxidising agent is molecular oxygen (O_2_), which thereby becomes chemically reduced to form hydrogen peroxide that is detected with an electrode (Equation (3)) [92].

(3) $$Glucose+O_{2}\to gluconolactone+H_{2}O_{2}\;$$

However, this measurement can be unstable if O_2_ levels fluctuate for any reason in the sample, e.g., in blood or tissue, or if another redox active species such as ascorbate is present. Therefore, some methods use another molecule as an artificial electron charge transfer moiety “mediator” [93], e.g., a ferrocene derivative (Fc), that acts preferentially as an oxidant with the glucose oxidase enzyme to “shuttle” electrons between the enzyme and the electrode—see Equation (4) [92]. This lowers the electrical potential for measuring the enzyme-catalysed reaction, thus diminishing the interference by redox active species present within the sample.

(4) $$Glucose+GOx_{\left(ox\right)}\to gluconolactone+GOx_{\left(red\right)}\;$$

$$GOx_{\left(red\right)}+2Fc^{+}\to GOx_{\left(ox\right)\;}+2Fc+2H^{+}\;$$

$$2Fc\;⇄\;2Fc^{+}+2e^{-}\;$$

In addition to TBI, it is worth mentioning the usefulness of combining microdialysis with biosensors in other disorders including diabetes mellitus and neurodegeneration. To better understand the neurochemistry of neurodegenerative diseases, Gunawardhana and Lunte have developed a separation-based sensor using microchip electrophoresis with electrochemical detection integrated with microdialysis for continuous measurements of adenosine and subsequent downstream metabolites [70]. Perrier and colleagues have also developed a microfluidic platinum black microelectrode array with microdialysis integration capabilities for real-time glucose monitoring in pancreatic islets [71]. 

**Figure 5:**
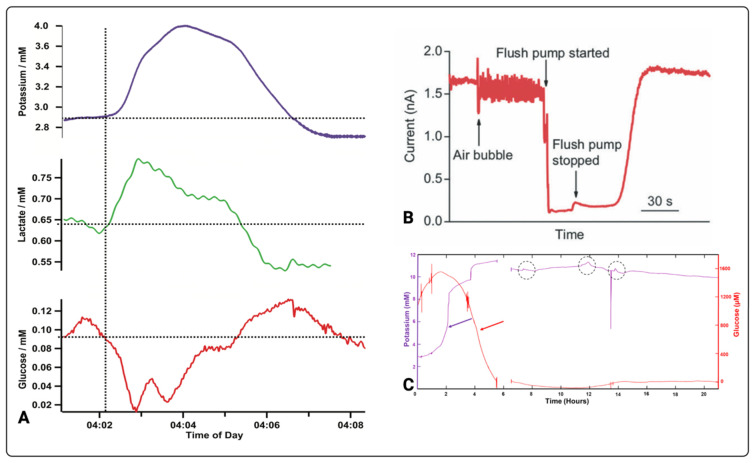
Brain metabolic changes measured by electrochemical biosensors. (**A**) Potassium, glucose, and lactate changes over time in a TBI patient [50]. (**B**) Microdialysate measurements affected by an air bubble [94]. (**C**) Simultaneous glucose and potassium changes over time in a patient 6 days after a TBI. The dotted circles indicate potassium transients while the red allows indicates glucose decline in relation to potassium rise (purple arrow) [51]. Images (**A**–**C**) originally appeared in [50], [94] and [51] respectively, published Open Access CC BY [50,94] and CC BY-NC-ND [51], copyright The Authors.

In TBI, most electrochemical sensors for neurochemical measurements are based on amperometric detection using specific enzymes that are selective to relevant brain metabolites. Papadimitriou and colleagues have developed an enzymatic-based sensor integrated with microdialysis for the amperometric detection of glucose [45]. Here, Papadimitriou et al. used glucose oxidase (Gox) and horseradish peroxidase (HRP) enzymes in 1.5 mM ferrocene monocarboxylic acid (Fc) solution that is immobilised onto a combined needle electrode within a microfluidic channel. The glucose-Gox reaction generates hydrogen peroxide that reacts with the mixture of HRP and Fc to form ferrocenium ions. This reduction process is quantifiable at the surface electrode, allowing the acquisition of glucose measurements. The system’s setup is displayed in Figure 3B, while technical details are communicated in Figure 4. Another enzymatic-based biosensor integrated with a microdialysis stream using a microfluidic PDMS chip by Pagkalos and colleagues has measured lactate concentration changes in vitro [46]. Here, they detected lactate levels between 0 and 50 µM with stepwise changes of 12.5 µM every 4 min. For continuous measurements, Robbins et al. reported below 10 µM extracellular glucose via dexamethasone-enhanced microdialysis coupled with an enzymatic-based sensor in ten rats [48]. Other examples of neurochemical measurements using electrochemical sensors have been presented in Table 2 and Figure 5.

A few studies have shown both glucose and lactate measurements in TBI using electrochemical sensors integrated with microdialysis. Tageldeen and colleagues have achieved both glucose and lactate measurements using wearable electrochemical sensors [47]. They were able to demonstrate dynamic metabolic changes ranging from 0 to 1 mM with 0.25 mM steps. Glucose and lactate calibration curves were produced using linear regression of R^2^ = 0.998 and R^2^ = 0.995, respectively, and the LoDs for glucose and lactate were 0.85 µM and 1.2 µM, respectively. 

Although in vitro studies enhance our understanding of neurochemical monitoring in TBI, their limitation in recapitulating the brain’s complex and dynamic changes means that simple non-biological model findings may not be replicated in humans. Continuously recorded microdialysis data in six patients for a period of over 6 h has shown that changes in the levels of glucose and lactate influence spreading depolarisations, as evidenced by increases in potassium levels [50]. In one of the patients (Figure 5A), they found basal glucose levels in the microdialysate to be low (100 µM) despite high blood glucose levels (11.8 mM) reflecting the brain tissue’s high energy demand, while a high lactate concentration is attributed to anaerobic glycolytic metabolism in the injured brain [50]. In another study, Gowers and colleagues also continuously monitored cerebral metabolites found in dialysates [94]. Here, they first tested their system in an in vitro brain model before testing it on patients. For non-clinical data, the sensor was continuously calibrated using physiological buffer and 2 mM glucose concentration with steps of 0.5 mM at a constant flow rate of 0.2 µL/min. The quality of the data acquired in a clinical setting was hindered by the air bubbles in the system leading to decreased signal-to-noise ratio (Figure 5B). However, following three hours of monitoring, the sensor detected a sudden lactate increase in one patient. Vital information such as this has also been presented by Gifford et al. (2021) in a follow-up study to the one previously discussed [48]. Here, Gifford and colleagues reported declining glucose levels in three TBI patients and persistent low glucose in one TBI patient. In one of the patients, glucose levels declined to non-detectable levels, as evidenced in Figure 5C. This long-term glucose decline has been said to be because of metabolic aberrations in an injured brain [51].

### 3.5. Optical Biosensors

Optical biosensors offer many advantages over conventional analytical methods owing to their direct, real-time, and label-free detection of many biochemical substrates. Optical detection works by exploiting the interaction of light with the analyte of interest or with a recognition element that is selective to the analyte. Various applications of optical sensing have been performed both in a label-free or label-based manner. For label-based detection, the optical signal is acquired by fluorescent or colorimetric techniques [95,96,97,98], whereas label-free techniques involve light–analyte interactions to facilitate signal detection exemplified by spectroscopic biosensors [49,64,65,66,67,68,69,99]. Other than the widely used offline ISCUSflex colorimetric bedside analyser, all optical based techniques integrated with microdialysis to quantify TBI metabolites have utilised infrared spectroscopic sensors.

Infrared spectroscopy has been successfully used for numerous applications in biochemistry as evidenced in the literature offering innovative diagnostic methods [99,100,101,102,103]. Specifically, mid-infrared (MIR) regions ranging from 2.5 to 25 µm has been primarily adopted for monitoring neurochemicals. Furthermore, investigators have opted for powerful mid-infrared light sources such as quantum cascade lasers (QCLs) for spectrometric analysis [49,64,99].

**Figure 6:**
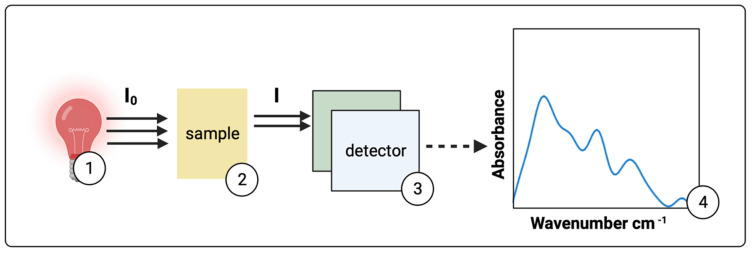
Basic principles of infrared spectroscopy. (**1**) Incident broadband infrared light (***I*_0_**) upon a (**2**) sample leads to vibrational modes of a molecule. Consequently, specific amounts of energy from the incident light are absorbed. This decreases the subsequently measured infrared light (***I***) by the detector (**3**). Data are then acquired for interpretation in the form of a spectrum expressed as absorbance versus wavenumbers in cm^−1^ (**4**).

Infrared spectroscopy allows the investigation of the interaction of light with organic compounds via the excitation of vibrational and rotational modes. The principle of measurement is shown in Figure 6, where infrared light passes through a sample containing the analytes of interest and MIR radiation interacts with microdialysate samples, providing a spectral fingerprint that is useful for identifying the molecules of interest such as glucose, lactate, and pyruvate. Supporting the absorbance analysis theory is the Beer–Lambert law [104] depicted in Equation (5).

(5) $$A=\log\left(\frac{1}{T}\right)=\varepsilon Lc\;$$

Here, *A* is absorbance, *T* is transmittance, ε is the molar absorptivity coefficient in L/((mol)(cm)) of the metabolite of interest at a certain wavelength, *L* is the path-length in cm through solution, and *c* is the concentration in molarity (M) of the metabolite of interest. Glucose, lactate, and pyruvate have varying absorbance intensities at specific wavelengths. The degree of absorption is determined by the wavelength of light, the absorption cross-section of the analyte, and the optical path-length.

**Figure 7:**
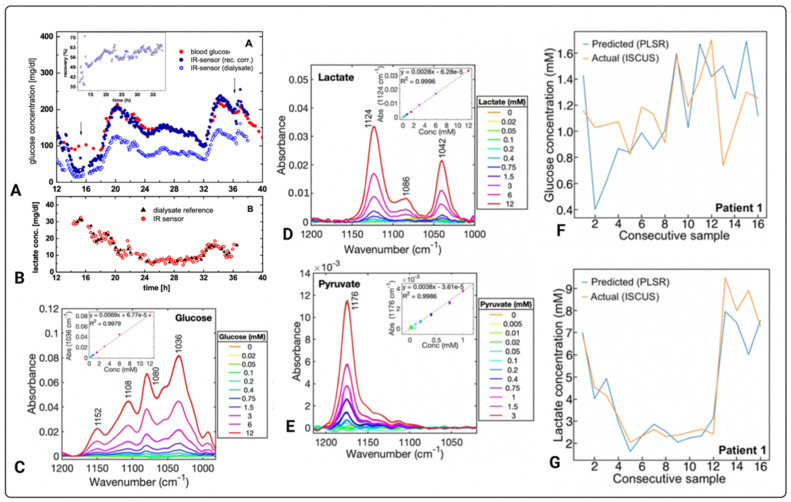
Measurements of various metabolites using optical biosensors. Glucose (**A**) and lactate (**B**) continuous measurements of concentration versus time, determined in a type 1 diabetic subject. The inset graph within (**A**) shows % recovery versus time [69]. The arrows within (**A**) indicate the time between which the tissue lactate concentrations in (**B**) were also followed. (**C**) Glucose, (**D**) lactate, and (**E**) pyruvate MIR measurements of absorbance vs. wavenumber (cm^−1^) for different concentrations of pure standards, whereby the insets are standard calibration curves of three replicates at specific wavelengths of each metabolite. (**F**,**G**) compare consecutively pooled microdialysate measurements of one TBI patient acquired by an optical based sensor to routinely used ISCUSflex microdialysis analyser where glucose and lactate was detected. The diagrams in (**A**,**B**) are published with permission of the Publisher and Corresponding Author*; they originally appeared in T. Vahlsing, S. Delbeck, J. Budde, D. Ihrig, and H. M. Heise*. Combination of micro-dialysis and infrared spectroscopy: a multi-analyte assay for accurate biofluid analysis and patient monitoring. Biomedical Vibrational Spectroscopy 2016: Advances in Research and Industry, edited by A. Mahadevan-Jansen, W. Petrich, Proc. of SPIE Vol. 9704, 97040R. © 2016 SPIE·CCC code: 1605–7422/16/$18, doi: 10.1117/12.2214636 [69]. Images (**C**–**G**) originally appeared in [49], published Open Access CC BY, copyright The Authors.

The versatility of optical sensing technologies integrated with microdialysis for reagent-free multi-analyte assays has been demonstrated by various studies [49,64,65,66,67,68,69,99]. Examples are presented in Figure 7 [49,69]. Heise and colleagues have developed an optical sensing system integrated with microdialysis for continuous measurements of analytes found in blood [65,66]. Using infrared transmission-mode, the system measured varying glucose concentrations between 5.2 and 15.8 mM for more than 90 h. Subsequently, they determined carbon dioxide and bicarbonate levels in blood to examine acid-base status of the body [64]. Other than ex vivo analysis of glucose levels in heparinised blood of type 1 diabetic patients [68], Vahlsing and colleagues have also simultaneously quantified glucose and mannitol in recovery-rate studies, as well as lactate, demonstrating the capabilities of the optical sensor to quantify relevant clinical substrates (Figure 7A,B) [69]. 

The developed infrared spectrometric sensor by Vahlsing and colleagues consists of a 30-micrometre pathlength transmission micro-cell with custom-built microfluidics that permits continuous automated sample measurements [69]. Figure 7A highlights the correlation and accuracy between a commercially available Beckman glucose analyser and their novel optical sensor in measuring blood glucose. Subsequently, lactate concentrations determined from the microdialysates spectra were compared against the reference lactate assay from 5 µL volumes of intermittently acquired dialysates. As shown in Figure 7B, as the lactate concentration normalises over time, the correlation of interstitial glucose to blood glucose is established [69].

As previously mentioned, advances in the field have led to the use of powerful light-sources to enhance accuracy, precision, and long-term stability when measuring relevant clinical metabolites [49,99]. Evidence shows that the use of powerful light sources, such as QCLs, instead of thermal emitters to quantify metabolites, yields better overall results [99]. Using a QCL-based spectrometric sensor integrated with microdialysis, Alimagham and colleagues quantified dialysates from two traumatically brain-injured patients acquired over the course of 48 h [49]. The analysis demonstrated the ability of the novel biosensor in detecting and measuring glucose and lactate in microdialysates of TBI patients. The biosensor exhibits LoDs of 0.5, 0.2, and 0.1 mM for glucose, lactate, and pyruvate, respectively. In vitro measurements of glucose, lactate, and pyruvate showed good linearity of the sensor, as shown in Figure 7C–E, respectively. Furthermore, highlighting the accuracy of the technology was the correlation of the results acquired using the QCL-based spectrometric sensor and the well-established enzymatic colorimetric assays on the ISCUSflex microdialysis analyser whereby glucose, lactate, and pyruvate levels were measured from microdialysates of two TBI patients ex vivo (Figure 7F,G).

## 4. Progress, Challenges, and Future Perspectives 

The sensing techniques reviewed here have shown promising results in providing metabolic insights of the injured brain. The enzymatic colorimetric method is used in the standard analyser for the quantification of the relevant TBI metabolites. High accuracy and precision in quantifying metabolic values including LPR justifies why it is the most reliable technique to date. However, hourly non-continuous measurements and the need for reagents are the main obstacles. Optical infrared spectroscopic sensors offer quick, precise, and reagent-free analysis, and this technique has shown promise in quantifying the brain metabolites in TBI. However, MIR technology’s feasibility to continuously monitor cerebral metabolites reliably online needs more evidence. Although electrochemical biosensors exhibit good analytical performance, their sensitivities gradually decrease with time when in use. Overall, this exhibits a challenge in recording the fluctuating metabolic concentrations often seen in severe TBI patients. Therefore, counteracting the gradual loss of sensitivities of these biosensors would facilitate high accuracy and reliable data acquired when monitoring the traumatically injured brain.

M Dialysis AB developed LOKE, a portable commercially available enzymatic-electrochemical sensor for continuous online clinical microdialysis monitoring. This automated biosensor relies on enzymatic detection to generate absolute glucose and lactate values, plus trends, and can run for up to 5 days [105]. The advantage for busy clinical staff is that LOKE eliminates the need for manual hourly assays on ISCUSflex. However, the detection of pyruvate is work in progress [105]. Once all three relevant brain metabolites become quantifiable, this would be a remarkable advance in the field. A limitation of LOKE is that it should only be used with microdialysis catheters with 20 kDa membrane pore size [105]. Boutelle’s group has already continuously monitored microdialysates online using home-built enzymatic-electrochemical-based sensors, and some of their findings have elucidated the pathophysiological changes in TBI patients, highlighting the utility of integrating biosensors with microdialysis [50,51,94]. However, some data acquired had noise and artefacts that contributed to the loss of vital information [94]. All biosensors mentioned in Table 2 are in the design and validation phase, with only work in references [50,51,94], testing their technologies on patients. Thus, there is a need for more validation studies in humans to establish their efficacy and robustness. Subsequently, these biosensors could be optimised for enhanced performance to achieve real-time continuous online microdialysis monitoring in TBI patients. Furthermore, implantable non-microdialysis sensors for direct monitoring of neurochemicals in the brain offer improved temporal resolution [106]. Booth and colleagues have measured lactate in the frontal and parietal cortex of mice using a fibre-based enzymatic sensor in situ [106]. Although in its infancy, the development of specific, ultra-sensitive, and stable probes for in vivo direct monitoring of brain metabolism will advance real-time brain monitoring of TBI patients. Therefore, more studies to further improve emerging techniques for clinical use are needed.

Future studies should also focus on the development of data ecosystems encompassing brain metabolism and other monitored parameters such as ICP and PBtO_2_ due to an interplay between cerebral metabolic demand and energy substrate delivery. For instance, brain oedema following TBI leads to an increase in ICP, which then causes a decrease in CPP, cerebral blood flow, and brain tissue oxygenation [107]. Due to this mismatch between cerebral metabolic demand and energy substrate delivery, the interpretation of brain chemistry should be in the context of the clinical condition of the patient and in conjunction with other monitored parameters including ICP, CPP, PBtO_2_, and cerebral vascular pressure reactivity (PRx) [16,108]. Thus, data acquired using biosensor technologies when incorporated with data from other monitored parameters can improve methods of monitoring real-time cerebral physiology to better understand secondary brain injury onset and treatment strategies. When used as a guide for medical intervention (Table 1), measurements acquired using these biosensors can aid in the prognostication of patient outcome. In addition, clinicians working in neuro-intensive care will benefit from improved clinical workflow facilitated by data ecosystems, allowing them to focus more of their time on direct patient care.

## 5. Conclusions

In this review, we have summarised biosensors coupled to microdialysis for the detection of cerebral metabolites in TBI. To date, electrochemical and optical sensors are the only types of biosensors used for cerebral metabolite measurements. The accuracy and actionable information provided by these emerging technologies can help inform clinical decision making as well as aid in the prognostication of patient outcome. Other than improved performances, feasibility and robustness of these biosensors can be enhanced by conducting in vivo clinical studies. Future designs of the biosensor technology will include ultra-sensitive and stable probes for in situ direct sensing of brain metabolites for true real-time brain monitoring in TBI patients.

## Figures and Tables

**Table 1:** Summary of neuroprotective interventions for altered cerebral metabolism [29,30,31,32,33,34,35,36,37,38,39,40,41,42,43,44].

Intervention	Effect	References
Glucose/insulin	↑↓ glucose, ↑↓ LPR	[29,30,31,32,33]
Hyperoxia	↑ PBtO_2_, variable ↓ LPR	[34,35,36]
Hyperventilation	↓ glucose	[37,38]
Mannitol	↓ LPR	[32,39]
Decompressive craniotomy	↓ LPR	[43,44]
Therapeutic (induced) hypothermia	↓ glucose, ↓ lactate	[41,42]

**Table 2:** A summary comparison of recent key examples of biosensors integrated with microdialysis for brain metabolite monitoring [45,46,47,48,49,50,51,52].

Study	Sensor Type	Setting	Comments
Papadimitriou et al. (2016) [45]	Enzymatic-electrochemical	In-vitro	Measured 0–100 μM glucose concentration, with 25 μM increments, in a microdialysate stream.
Pagkalos et al. (2018) [46]	Enzymatic-electrochemical	In-vitro	Measured 0–50 μM lactate concentrations with 12.5 μM increments using enzymatic based sensor with LoD range 2.5 to 9.5 nM, in a microdialysate stream.
Tageldeen et al. (2020) [47]	Enzymatic-electrochemical	In-vitro	Measured 0–1 mM glucose and lactate, changing concentrations. LoDs of 0.85 and 1.3 μM for glucose and lactate, respectively, in a microdialysate stream.
Robbins et al. (2019) [48]	Enzymatic-electrochemical	In-vivo (rats)	Reported progressive decrease in glucose in microdialysates from a cortical impact injury.
Rogers et al. (2017) [50]	Enzymatic-electrochemical	In-vivo (human)	Continuous online microdialysis measurements in TBI patients; monitoring duration > 6 h; glucose, lactate, and K^+^ levels in spreading depolarisation (K^+^ was measured by an ion-selective electrode).
Gowers et al. (2019) [52]	Enzymatic-electrochemical	In-vivo (human)	Detected a sudden surge of lactate levels during continuous online dialysate measurements in TBI patients.
Gifford et al. (2021) [51]	Enzymatic-electrochemical	In-vivo (human)	Reported declining glucose levels in 3 TBI patients, and persistent low glucose in 1 TBI patient, in dexamethasone-enhanced continuous online microdialysis.
Alimagham et al. (2021) [49]	Optical (mid-IR)	Ex-vivo (human)	Microdialysate measurements from TBI patients, offline. LoDs of 0.5, 0.2, and 0.1 mM for glucose, lactate, and pyruvate respectively. Quantification of brain metabolites was compared with a conventional enzymatic-colorimetric microdialysis analyser (ISCUSflex).

**Table 3:** Important sensor parameters in the context of brain metabolism.

Parameter	Meaning
Selectivity	The sensor should be able to detect a molecule of interest, e.g., glucose, in the presence of other molecules and endogenous substances found in microdialysates.
Sensitivity	The sensor should be able to detect the relevant range of metabolite concentrations seen in TBI.
Stability	The sensor should not be influenced by changes in the external or internal environment when monitoring brain metabolism as this can lead to distortion of output signals.
Linearity	The sensor should measure accurate concentration changes proportionately, and ideally in a linear manner.
Reproducibility	The results acquired by the sensor should be of highest accuracy and the investigator should be confident of achieving the same results if the brain metabolic conditions were constant.
Multiplexing	Ability of the sensor to detect several analytes simultaneously, e.g., glucose, lactate, and pyruvate, in microdialysates containing these and other endogenous molecules.

## Abbreviations

TBI	Traumatic brain injury
ICP	Intracranial pressure
LPR	Lactate pyruvate ratio
PBtO_2_	Brain tissue oxygen
ATP	Adenosine triphosphate
TCA	Tricarboxylic acid
PPP	Pentose phosphate pathway
NAD^+^	Nicotinamide adenine dinucleotide (oxidised)
NADH	Nicotinamide adenine dinucleotide (reduced)
FAD	Flavin adenine dinucleotide (oxidised)
FADH_2_	Flavin adenine dinucleotide (reduced)
LoD	Limits of detection
GOx	Glucose oxidase
MIR	Mid-infrared
CNS	Central nervous system
CPP	Cerebral perfusion pressure
PRx	Pressure reactivity index
